# Capacity and Readiness Assessment of Healthcare Facilities for Digital Health Interventions Against Tuberculosis and HIV in Addis Ababa, Ethiopia

**DOI:** 10.3389/fdgth.2022.821390

**Published:** 2022-02-28

**Authors:** Emnet Getachew, Yimtubezinash Woldeamanuel, Tsegahun Manyazewal

**Affiliations:** ^1^Center for Innovative Drug Development and Therapeutic Trials for Africa (CDT-Africa), College of Health Sciences, Addis Ababa University, Addis Ababa, Ethiopia; ^2^Department of Public Health, College of Health Science, Arsi University, Asella, Ethiopia

**Keywords:** digital health, eHealth, health technology, tuberculosis (TB), human immunodeficiency virus (HIV), Ethiopia

## Abstract

**Background:**

There is a high level of concern that low-income countries lack the capacity and readiness to effectively adopt, implement, and scale up digital health interventions (DHIs). We aimed to assess the infrastructure and human resource capacity and readiness of healthcare facilities to adopt and implement any new DHI for tuberculosis (TB) and HIV care and treatment in Addis Ababa, Ethiopia.

**Method:**

We carried out a cross-sectional, mixed-methods study in 14 public healthcare facilities that provide TB and HIV care and treatment services. Providers' perceived readiness to adopt and implement digital health was assessed using a self-administered questionnaire designed based on an adapted eHealth readiness assessment model that covers six domains: core readiness, organizational cultural readiness, value proposition readiness, technological readiness, regulatory policy readiness, and operational resource readiness. The infrastructure and human resource capacity were assessed on-site using a tool adapted from the Technology Infrastructure Checklist. Internal consistency was assessed using Cronbach's alpha, and the significant relationship between the composite variables was assessed using Pearson's correlation coefficients (*r*).

**Result:**

We assessed 14 facilities on-site and surveyed 60 TB and HIV healthcare providers. According to *Cronbach's alpha test*, all the six technology acceptance domains had a value of >0.8, suggesting a strong interrelatedness between the measuring items. The correlation between technological readiness and operational resource readiness was significant (*r* = *0.8*). The providers perceived their work environment as good enough in electronic data protection, while more efforts are needed in planning, training, adapting, and implementing digital health. Of the 14 facilities, 64.3% lack the plan to establish a functional local area network, and 43% lack skilled staff on payroll to provide maintenance of computers and other digital technologies.

**Conclusion:**

Like many developing countries, there was a modest infrastructure and human resource capacity and readiness of public healthcare facilities in Addis Ababa, Ethiopia, to nurture and strengthen DHIs across the TB and HIV cascades of care. Technological and operational resource readiness, including funding and a Well-trained workforce, are essential for successful implementation and use of digital health against the two infectious diseases of global importance in such settings.

## Introduction

Digital health interventions (DHIs) are specified as using digital devices, mobile and wireless technologies to support the achievement of health goals (1–3). The World Health Organization (WHO) defines DHI as a discrete functionality of digital technology applied to attain health objectives (4). DHI indicates the general use of necessary information and communication technologies (ICT) for health, consisting of both mobile health (mHealth) and electronic health (eHealth). Various countries have been evaluating the potential uses of DHI to improve treatment adherence, medical records, disease surveillance, program monitoring, treatment follow-up, laboratory management, and eLearning to enhance clinical care, treatment, and disease prevention and control (5–9).

The main challenge to implementing and integrating DHIs into the health system for evidence-based decision-making is the lack of enough information about country-specific digital health capacity and the larger ICT ecosystem (10–13). Understanding how theoretically promised DHIs to work within a specific local context is significant to ascertain context-sensitive DHIs implementation and scale-up. Information about the legal, ethical, and social implications in the adoption of DHIs and where and how such technologies can be deployed have been a major gap in the literature (14–16). Acceptability of such technologies by front-line healthcare providers is one of the indicators of health facility readiness to adopt and implement DHIs. The theoretical basis for this study is that healthcare workers' perspectives and the health facilities' capacity and readiness are critical in determining the extent and success of the implementation of DHIs.

There have been decades of investments made in the prevention and control of tuberculosis (TB) and human immunodeficiency virus (HIV), the two infectious diseases of global importance. Several studies have confirmed that TB and HIV will continue to pose major challenges without the deployment of innovative prevention and treatment strategies for everyone who needs them (17–21). For countries like Ethiopia that are overwhelmed by the dual burden of the two diseases, advancing the diagnosis, care, and treatment programs are essential to meeting global targets such as the 2035 End TB Strategy and the 2030 end AIDS epidemic (22–26). The Use of DHIs could be one of such elements while understanding the current infrastructure, human resources, and health systems potential is critical for their successful deployment and practice.

Thus, this study aimed to assess the infrastructure, and human resource capacity, and readiness of healthcare facilities to adopt and implement any new DHI for tuberculosis (TB) and HIV care and treatment in Addis Ababa, Ethiopia.

## Methods

A facility-based, mixed-method cross-sectional study was conducted in government-owned hospitals and health centers (*n* = 14) in Addis Ababa, Ethiopia. The study used a combination of an interview with a semi-structured, interviewer-administered questionnaire, self-administered questionnaire, and a checklist-based assessment of sites. Data were collected between January and March 2021.

### Setting and Participants

There were 94 health centers in the 10 sub-cities of Addis Ababa, of these, 10 with high TB/HIV patient flow, one from each sub-city, were included (Table 1). There were six hospitals led by the city administration, of these, four with high TB/HIV patient flow were included.

**Table 1:** Included public health centers from each sub-city.

**No**.	**Name of Health Center**	**Sub-city**
1	Addis Raey Health Center	Addis ketema
2	Akaki Health Center	Akaki kality
3	Kebena Health Center	Arada
4	Goro Health Center	Bole
5	Adisu Gebeya Health Center	Gulele
6	Kazanchis Health Center	Kirkos
7	Alem Bank Health Center	Kolfe
8	Teklehaymanot Health	Lideta
9	Woreda 02 Health Center	Nifasilk lafto
10	Woreda 13 Health Center	Yeka

The study population was all TB or HIV healthcare providers who were working in the 14 selected healthcare facilities. The study included consented healthcare professionals who were working in TB or HIV clinics at the time of data collection, but excluded those with <6 months of work experience as they may have little or ambiguous information about the study site and the subject of interest. The sample size depended on the scope of the study and the possibility of getting eligible participants in each study site; thus guided by the purposive sampling technique. There were 76 healthcare providers working at the selected sites, of whom 60 (80%) met the inclusion criteria and participated.

### Data Collection

**Figure 1:**
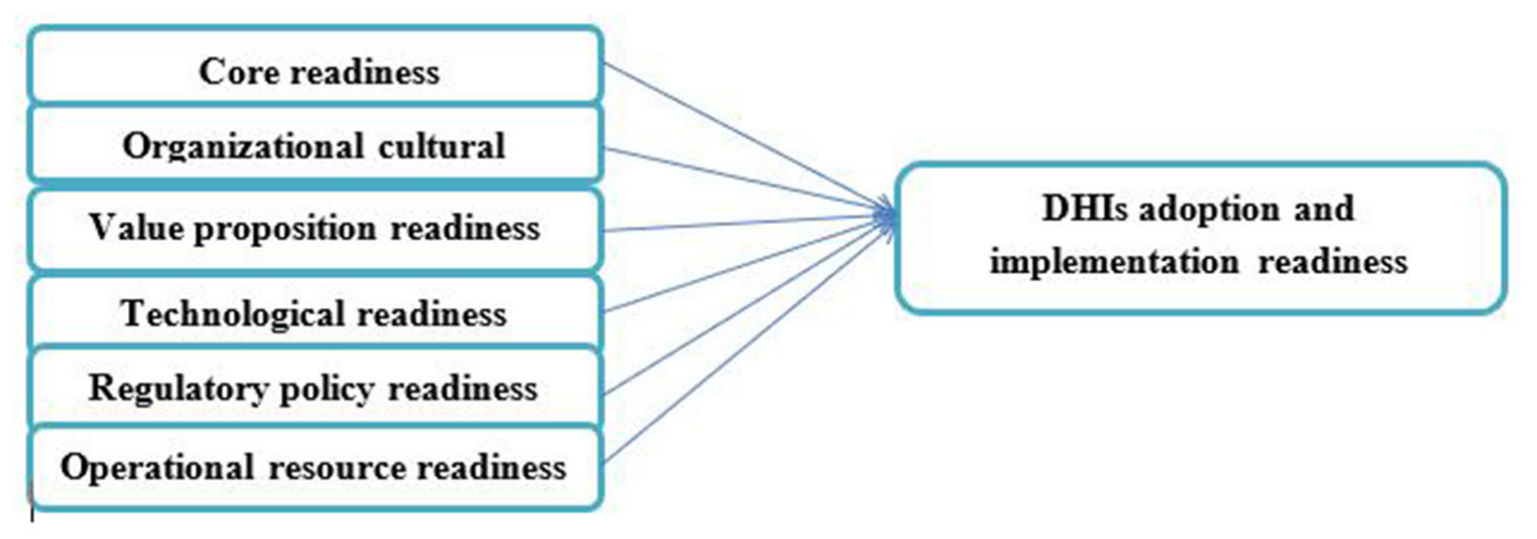
DHIs Adoption and Implementation Readiness model (27–29).

The participants responded to a self-administrated, structured, and adopted questionnaire aimed to assess the facilities' capacity and readiness to adopt and implement DHIs. The questionnaire was adapted from eHealth readiness assessment models (27–29) that cover six domains: core readiness (CR); organizational cultural readiness (OCR); operational resource readiness (ORR); technological readiness (TR); value proposition readiness (VPR); and regulatory and policy readiness (RPR) (Figure 1) (27–29).

The questionnaires were filled out by a person who was entitled to a managerial position in each department, i.e., HIV and TB clinics, to assess facility readiness (*n* = 28). A semi-structured interview was also conducted, and the interview was mainly focused on the previous experience of care providers in using different technologies and their overall evaluation of their facilities' readiness to adopt and implement new digital technology in their department (*n* = 60). The healthcare providers usually work in very busy clinics; therefore, nearby unoccupied office spaces were used to ensure participants' privacy and to manage their time effectively. The interview was lasted for about 20 to 30 min and was conducted in the local language (Amharic) or English depending on the interest of each participant, and it was audio recorded. The audio recording was later transcribed and the Amharic translated to English to produce de-identified English-language transcripts.

A checklist adapted from the Technology Infrastructure Checklist and other sources (30, 31) was used to assess the current infrastructure and human resource capacity of the included facilities, with emphasis given to TB and HIV clinics. The purpose was to understand the gaps and opportunities to adopt new DHIs in those sectors. The checklist was completed by making on-site observations, and if any additional information was needed, facility-level health information managers were consulted.

### Data Processing and Management

Any physical records including informed consent forms and paper-based questionnaires were safeguarded in a locked personal cabinet. Interview records and transcripts were stored on a coded password-protected computer to ensure the confidentiality of participants' data. One-quarter of English language transcripts were randomly chosen and were assessed against the original audio recordings to verify the correctness and completeness of the gathered data.

### Data Analysis

The close-ended questionnaire and the checklist were analyzed quantitatively using SPSS version 20, and most interview questions were analyzed qualitatively using thematic analysis. All readiness assessment variables had a 5 point Likert scale value. Depending on the questions, the responses were dichotomized. The Likert scale questions with potential responses “No never considered,” “No but have considered,” “Yes in progress,” “Yes nearly completed,” and Yes in place” were analyzed considering the last three responses supportive that indicate positive values. Similarly, the Likert scale questions with the response “Agree” and “Strongly agree” were aggregated as positive values.

### Data Quality Assurance

The reliability and internal consistency of each factor in the adapted data collection instrument were assessed using Cronbach's Alpha, with the value 0.7 used as the cut-off point. A Pre-test of the study questionnaires was conducted at selected health facilities on 10% of the estimated sample size. The training was provided to the study data collectors. A Study Supervisor and the Principal Investigator verified the collected data for completeness daily.

### Ethical Considerations

The study protocol has been reviewed and approved by the Scientific and Ethics Review Committee of the Center for Innovative Drug Development and Therapeutic Trials for Africa (CDT-Africa), College of Health Sciences, Addis Ababa University, and the Ethics Review Committee of the Addis Ababa Health bureau. An official letter was sent to each study site for permission to undertake the study accordingly. At the individual level, after a clear explanation of the purpose and importance of the study, written informed consent was obtained from all participants before they participate in the study.

## Result

### Socio-Demographic Characteristics

We surveyed 60 TB and HIV healthcare providers, of whom 60% were female, 42% were aged between 31 and 40 years, 65% held a BSc degree, and 36.7% had more than 10 years of working experience (Table 2). Departmentally, 62% were working in HIV clinics.

**Table 2:** Socio-demographic characteristics.

	**No**.	**%**
**Total**	60	100
**Gender**		
Male	24	40
Female	36	60
**Age**		
18–30	16	26.7
31–40	25	41.7
41–50	15	25
Above 51	4	6.7
**Educational level**		
College diploma	10	16.7
BSc	39	65
MSc	11	18.3
**Department**		
TB room	23	38.3
HIV room	37	61.7
**Work experience**		
>1 year	2	3.3
2–5	16	26.7
6–9	20	33.3
Above 10	22	36.7

### Technology Utilization

Eighty percent of the participants have been using DHIs in their respective facilities. Healthcare providers at HIV clinics utilized a smart care system to keep patient data electronically, report data to concerned bodies, and retrieve data. The healthcare providers had been given some training to use such technologies appropriately, while only 26% were satisfied with the training, replying that the training provided was not enough to use the technology appropriately (Table 3).

**Table 3:** Responses of leading questions by the respondents (*n* = 60).

**Leading questions**		**Percent of cases**
Q1	HCPs heard of DHIs	29 (48.3%)
	HCPs with smartphone	51 (85%)
	Willingness to use various technologies in the facility	54 (90%)
	Computer access in the healthcare facility	33 (55%)
	HCPs having different online training	7 (11.7%)
	Experience using EMR	37 (61.7%)
	Experience in using any other technologies for TB/HIV patients	48 (80%)
	The relative advantage of technology	45 (75%)
	The simplicity of the technology	42 (70%)
	Related training that would help to implement such technology	41 (68.3%)
	Adequacy of the training	16 (26.7%)
	Favorable environment or infrastructure	26 (43.3%)
	Challenges to use DHIs in the facility?	52 (86.7%)
	Opportunities in the facility to adopt new DHIs?	49 (81.7%)

According to the data obtained from the respondents, TB care providers in hospitals were less familiar with DHI utilization than those in health centers. TB care providers were in the process to involve in a pilot study that will be using Digital Adherence Technologies (DATs) for adherence to TB treatment and that they were trained already and given the necessary information technology supplies. Among the respondents, 43% confirmed that they had a favorable working environment to utilize new DHIs in their facilities, and 82% endorsed sundry opportunities in the health facilities to properly implement digital health interventions in both health centers and public hospitals under the study.

### Facility Readiness to Adopt and Implement New DHIs

All study facilities had a dedicated annual budget, a procedure to secure patients' confidentiality and deliver services using DHIs, while only one facility implemented a multi-use system (connectivity among HCPs, laboratory, and others) and only three (21%) had governmental and institutional DHI policies in place to use and abided by Table 4.

**Table 4:** Results from the checklist that assessed facilities' infrastructure and human resource capacity.

**Description**	**Frequency**	**Percent**
Personnel (professional IT staffs)	14	100
Are hardware and software required for healthcare deliveries readily available?	9	64.3
The dedicated annual budget for improving IT	14	100
Is there any external consultant for installation and maintenance?	8	57.1
Is there establish inputs from leadership/management for sustaining the system?	9	64.3
Any currently delivered services using technology?	14	100
Any multi-user system (connectivity among HCPs, laboratory, and others)	1	7.1
Is there a procedure to secure patients' confidentiality?	14	100
Are governmental and institutional policies being in place to promote and manage the use of DHIs?	7	50

Most of the healthcare providers had intermediate computing and application skills, with 13.3% having fundamental skills in typing and using mouse and 3.3 having advanced computing and application (Table 5).

**Table 5:** Computer skills of HCPs.

**Computer skill of HCPs**	**Frequency**	**Percent**
Fundamental (typing and using mouse)	8	13.3
Basic computing and application	16	26.7
Intermediate computing and application	34	56.7
Advanced computing and application	2	3.3
**Total**	**60**	**100.0**

Data of the respondents showed that 57% of facilities had skilled staff on payroll for maintaining computers and other dysfunctions related to technologies. We observed the average number of computers in each facility during data collection; it was about 20 from the sampled healthcare facilities, with 71% of the facilities having more than 20 computers. However, for TB and HIV clinics, the average number of computers was not more than 10. Most of the participants used Wi-Fi for service provision, but 43% used Wi-Fi and broadband internet (cable). Thirty-six percent of the facilities had a plan to establish a functional Local Area Network (LAN) for interconnectivity to give better services (Table 6).

**Table 6:** Checklist on the infrastructure and human resource.

**Description**		**Frequency**	**Percent**
Staff with computing skills	Data entry	2	14.3
	Database mgmt.	4	28.6
	Having all skills	8	57.1
	**Total**	**14**	**100**
How does your office manage computing	Outsource whenever necessary	4	28.6
equipment maintenance?	Using skills of staff on payroll	8	57.1
	No Maintenance or irregular	2	14.3
	**Total**	**14**	**100**
Number of computers	Below 20	4	28.6
	Above 20	10	71.4
	Total	14	100
Internet access	Wi-Fi	8	57.1
	Both Wi-Fi and cable	6	42.9
	**Total**	**14**	**100**
Do you have a functional	NO	6	42.8
Local Area Network for	Yes	1	7.1
interconnectivity for a general use?	plan to establish	5	35.7
	**Total**	**14**	**100**
How do you ensure security for computing equipment?	Using the resident guard/police	14	100

### DHIs Adoption and Implementation

*Cronbach's alpha* values for core readiness and organizational cultural readiness were 0.803 and 0.813, respectively. For value proposition readiness, technological readiness, Regulatory policy readiness, and Operational resource readiness, Cronbach's alpha value was found to be 0.837, 0.880, 0.905, and 0.871, respectively. Such values suggest a strong interrelatedness between measuring items.

Core readiness assessments are meant to guide development efforts by providing benchmarks for comparison and appraising progress. Digital health interventions readiness process based on an objective assessment leads to sound strategies that can offer a path for transfiguring good intentions into planned action that brings real change to people's lives. Using this theme, we had assessed the strategic planning of the facility. Surprisingly, only 32% of the respondents replied positive responses regarding their need assessment plan on DHIs.

Eighty-two percent of the given health facilities identified other interested health facilities collaborators and stakeholders. Based on this, ICAP and AHF (AIDS Healthcare Fund) were stakeholders in collaborating and facilitating technology utilization. These institutions mainly focused on HIV clinics, provided computers and training for the facilities and HCPs, and 58% of respondents believed that their management or leadership had supported DHIs initiative.

Sixty-eight percent of the respondents believed that DHIs support the care delivery mission of their respective facilities. Furthermore, 93% responded that there was a system or process to assure patients' safety and confidentiality. On the other hand, only 39% of respondents reported that care providers in the facilities were licensed/being licensed/trained to provide care through DHIs. Forty-nine percent of the respondents identified the medical requirements that have met the standards for properly implementing the DHIs in health facilities. Similarly, 46% confirmed that the facilities had examined the DHIs implemented in the context of workflow in their respective facilities.

There was an absence of guidelines on how to use DHIs in healthcare facilities. Participants blamed and critiqued the lack of any Digital Health policy document, which they understood was hampering the ability of responsible agencies to conduct and coordinate the activities of various existing silos of digital health-related projects in the facilities. The process of ensuring the availability of relevant tools for DHIs usage for both care providers and care receivers/patients had been identified only by 39% of the respondents. However, 79% of respondents confirmed an excellent collaboration with IT staff to implement DHIs in the facility. Overall, most of the respondents believed that more needs to be done for IT to be fully recognized as an essential tool to improve the quality of healthcare delivery. The majority, i.e., 75%, believed legitimate reasons to introduce a computer-based system in their unit, and 93% needed new tools to improve their workaround. Only 25% of respondents approved that their respective facility is ready to adopt and implement DHIs to advance healthcare delivery (Table 7).

**Table 7:** Facilities readiness (*n* = 28).

**Item**		**Frequency**	**Percent**
**Core Readiness**			
The facility conducted prior	No never considered	12	42.9
DHI needs assessment	No, but have considered	7	25
	Yes, in progress	7	25
	Yes, nearly completed	2	7.1
	**Total**	**28**	**100**
The facility has a plan to	No never considered	12	42.9
adopt technologies	No, but have considered	6	21.4
	Yes, in progress	8	28.6
	Yes, nearly completed	2	7.1
	**Total**	**28**	**100**
**Organizational Cultural Readiness**			
End users involved in	No never considered	14	50
planning process	No, but have considered	9	32.1
	Yes, rarely participated	1	3.6
	Yes, participated	4	14.3
	**Total**	**28**	**100**
The facility identified	Strongly disagree	1	3.6
collaborators.	Disagree	4	14.3
	Neural	5	17.9
	Agree	7	25
	Strongly agree	11	39.3
	**Total**	**28**	**100**
DHI initiatives supported	Strongly disagree	5	17.9
by management	Disagree	4	14.3
	Neural	2	9.8
	Agree	10	33
	Strongly agree	7	25
	**Total**	**28**	**100**
**Value proposition readiness**			
Existing DHIs support care	Strongly disagree	3	10.7
delivery mission	Disagree	3	10.7
	Neural	3	10.7
	Agree	8	28.6
	Strongly agree	11	39.3
	**Total**	**28**	**100**
Patients' safety assurance	Strongly disagree	0	0
is in place	Disagree	1	3.6
	Neural	1	3.6
	Agree	9	32.1
	Strongly agree	17	60.7
	**Total**	**28**	**100**
**Technological readiness**			
Practical viability DHIs	No never considered	7	25
checked	No, but have considered	4	14.3
	Yes, in progress	10	35.7
	Yes, nearly completed	4	14.3
	Yes, in place	3	10.7
	**Total**	**28**	**100**
Facility examined the DHIs	No never considered	10	35.7
to be implemented	No, but have considered	5	17.9
	Yes, in progress	5	17.9
	Yes, nearly completed	6	21.4
	Yes, in place	2	7.1
	**Total**	**28**	**100**
Guideline on the use of	No never considered	12	42.9
technology available	No, but have considered	5	17.9
	Yes, on process/ in progress	6	21.4
	Yes, nearly completed	3	10.7
	Yes, in place	2	7.1
	**Total**	**28**	**100**
Patient data protection	No never considered	2	7.1
measures are in place	No, but have considered	5	17.9
	Yes, on process/in progress	8	28.6
	Yes, nearly completed	7	25
	Yes, in place	6	21.4
	**Total**	**28**	**100**
**Operational resource readiness**			
DHI tools for providers and	No never considered	11	39.3
patients identified	No, but have considered	6	21.4
	Yes, in progress	6	21.4
	Yes, nearly completed	3	10.7
	Yes, in place	2	7.1
	**Total**	**28**	**100**
There exist good	Strongly disagree	1	3.6
collaboration with IT staffs	Disagree	2	7.1
to implement DHIs	Neural	3	10.7
	Agree	9	32.1
	Strongly agree	13	46.5
	**Total**	**28**	**100**
**Digital health interventions readiness**			
Legitimate reasons exist to	Strongly disagree	1	3.6
introduce computer-based	Disagree	3	10.7
system in the TB/HIV unit	Neutral	3	10.7
	Agree	10	35.7
	strongly Agree	11	39.3
	**Total**	**28**	**100**
Staff need new tools to	Disagree	1	3.6
improve the work	Neutral	1	3.6
	Agree	9	32.1
	Strongly agree	17	60.7
	**Total**	**28**	**100**
Staff in TB/HIV unit will	Disagree	2	7.1
benefit from DHIs	Neutral	1	3.6
	Agree	16	57.1
	Strongly agree	9	32.1
	**Total**	**28**	**100**
DHIs contribute to TB/HIV	Disagree	2	7.1
unit's performance	Agree	5	17.9
	Strongly agree	21	75
	**Total**	**28**	**100**
The facility is ready to	Strongly disagree	1	3.6
adopt and implement DHIs	Disagree	6	21.4
	Neutral	5	17.9
	Agree	9	32.1
	Strongly agree	7	25
	**Total**	**28**	**100**

### Correlation Analysis

A Pearson product-momentum correlation (Pearson *r*) was conducted to assess any significant relationship between the composite variables. The Pearson correlation coefficient quantifies the strength of a linear association between two variables and is denoted by *r*. The variables were measured on a ratio scale, which is a prerequisite for using Pearson correlation. All variables demonstrated a positive relationship, i.e., *r* values were all positive in the range of 0.4 to 0.8 and *p* < 0.05 for all, suggesting the contribution of these composite variables toward the assessment of DHIs adoption readiness of selected healthcare facilities, as a dependable construct. The *r-*value, the correlation between Technological Readiness and Operational Resource Readiness was 0.8. As a result, the correlation between Technological Readiness and Operational Resource Readiness was strongly significant in this analysis (Table 8).

**Table 8:** Correlations analysis.

		**Core readiness**	**Operational resource readiness**	**Value proposition readiness**	**Technological readiness**	**Regulatory policy readiness**	**Organizational cultural readiness**
Core readiness	Pearson Correlation	1	0.550^**^	0.397^*^	0.543^**^	0.401^*^	0.465^*^
Operational resource readiness	Pearson Correlation	0.550^**^	1	0.404^**^	0.801^**^	0.538^**^	0.486^**^
Value proposition readiness	Pearson Correlation	0.397^*^	0.404^**^	1	0.528^**^	0.419^*^	0.514^**^
Technological readiness	Pearson Correlation	0.543^**^	0.801^**^	0.528^**^	1	0.630^**^	0.675^**^
Regulatory policy readiness	Pearson Correlation	0.401^*^	0.538^**^	0.419^*^	0.630^**^	1	0.576^**^
Organizational cultural readiness	Pearson Correlation	0.465^*^	0.486^**^	0.514^**^	0.675^**^	0.576^**^	1

* *Correlation is significant at the 0.05 level (2-tailed)*.

** *Correlation is significant at the 0.01 level (2-tailed)*.

## Discussion

Many have argued that digital health promises enhanced efficiency and quality in healthcare. A facilitating factor for adopting and successfully implementing digital health is the acceptance and readiness of healthcare providers. Based on our findings, all healthcare facilities had professional IT staff and a dedicated annual budget to improve the IT department. All of them currently deliver services using different technologies and had a procedure to protect and keep patients confidentiality. The healthcare facilities used different technologies to simplify their service provision to patients and report and archive data.

Our findings showed that about half of the respondents had limited experience in computing and application skills, and with basic computer skills. Previous studies indicate that healthcare providers with good computer skills or competent enough in using computers were more likely to express their readiness for DHIs implementation and adopt the system in their facilities (32–34). Given the low computer skill of healthcare providers in developing countries, the findings call for a refresher or basic computer skill-based training to be given to healthcare providers before the implementation of DHIs.

In the current study, most care providers mentioned their readiness to use different technologies over the traditional method. They had confirmed the availability of internet access (Wi-Fi and broadband internet), the readiness of generators during light interruption, and accessibility of computers as a good opportunity to implement different technologies currently and in the future. On the contrary, the poor signal strength, lack of maintenance, in some facilities shortage of computers, and above all an absence of adequate spaces were mentioned as the major gaps to effectively implement DHIs. Inadequate training sessions to effectively implement such technologies was also judged a major gap. On the contrary, the willingness of care providers to use technologies and the willingness and potential support of facility leadership were promising enablers of DHIs. The capacity and effective utilization of digital health among healthcare workers depend on their workload, time spent, and documentation length, indicating that the training methods should meet the healthcare providers' learning needs (35).

According to the current study, only one facility established LAN for general use, while the rest 13 facilities use interconnectivity only for HIV units. One-third of the facilities had a plan to establish LAN for general use, with potential interconnecting departments and units across the facility. Most of the healthcare facilities have been looking for DHIs that would improve their service delivery and a significant number of healthcare providers agree that DHIs do support the healthcare delivery assignment of their facilities. Key technological categories are also needed to support the implementation process. However, the inadequacy of legislation and policies impeded the implementation of DHIs during the roll-out of large-scale DHIs to navigate the complexities of achieving the milestone and ensuring sustainability (36).

Currently, evidence is insufficiently available on the effectiveness of using DHIs to improve TB and HIV management (37–41). For Ethiopia, where the two diseases are yet the major disease burdens (42–45), studies on DHIs are desperately needed. Be it in hospitals or health centers, implementation of new DHIs in the study facilities require ICT platforms, dedicated and skilled staffing, leadership support, sustainable financing, and stakeholders engagement. The study findings can serve as a baseline for other researchers who would like to do more studies in the field of digital health and can guide policy-makers to intervene in the ongoing implementation of DHIs. Digital health is a new field of study that this study intended to look into. However, more studies are needed that go beyond Addis Ababa to provide a general picture of the finding in Ethiopia. The study assessed the capacity and readiness of healthcare facilities for DHIs against TB and HIV, thus worth extending the assessment for other diseases of national and global importance.

## Conclusion

Like many developing countries, there was a modest infrastructure and human resource capacity and readiness of public healthcare facilities in Addis Ababa, Ethiopia, to nurture and strengthen DHIs across the TB and HIV cascades of care. Technological and operational resource readiness, including funding and a Well-trained workforce, are essential for successful implementation and use of digital health against the two infectious diseases of global importance in such settings.

## Data Availability Statement

The raw data supporting the conclusions of this article will be made available by the authors, without undue reservation.

## Ethics Statement

The studies involving human participants were reviewed and approved by the Scientific and Ethics Review Committee of the Center for Innovative Drug Development and Therapeutic Trials for Africa (CDT-Africa), College of Health Sciences, Addis Ababa University, and the Ethics Committee of Addis Ababa Health bureau. The participants (healthcare proviers in TB and HIV departemets) provided written informed consent to participate in this study.

## Author Contributions

EG: study conception, data acquisition, synthesis, and first draft. TM and YW: data acquisition and synthesis. TM: resource acquisition. All authors reviewed and approved the final version for publication.

## Funding

This study was supported by the Center for Innovative Drug Development and Therapeutic Trials for Africa (CDT-Africa), College of Health Sciences, Addis Ababa University. TM was supported in part by the Fogarty International Center and the National Institute of Allergy and Infectious Diseases of the US National Institutes of Health (D43TW009127).

## Author Disclaimer

The content is solely the authors' responsibility and does not necessarily represent the official views of the CDT-Africa or the National Institutes of Health.

## Conflict of Interest

The authors declare that the research was conducted in the absence of any commercial or financial relationships that could be construed as a potential conflict of interest.

## Publisher's Note

All claims expressed in this article are solely those of the authors and do not necessarily represent those of their affiliated organizations, or those of the publisher, the editors and the reviewers. Any product that may be evaluated in this article, or claim that may be made by its manufacturer, is not guaranteed or endorsed by the publisher.
